# A gradient of matrix-bound FGF-2 and perlecan is available to lens epithelial cells^☆^

**DOI:** 10.1016/j.exer.2013.12.004

**Published:** 2014-03

**Authors:** Weiju Wu, Frederique M. Tholozan, Martin W. Goldberg, Leon Bowen, Junjie Wu, Roy A. Quinlan

**Affiliations:** a Biophysical Sciences Institute, School of Biological and Biomedical Sciences, Durham University, South Road, Durham DH1 3LE, United Kingdom; b NETPark Incubator, Thomas Wright Way, Sedgefield, Durham TS21 3FD, United Kingdom; c Biophysical Sciences Institute, Department of Physics, Durham University, Durham DH1 3LE, United Kingdom; d Biophysical Sciences Institute, School of Engineering and Computing Sciences, Durham University, Durham DH1 3LE, United Kingdom

**Keywords:** lens capsule, FGF-2, perlecan, ERK1/2

## Abstract

Fibroblast growth factors play a key role in regulating lens epithelial cell proliferation and differentiation via an anteroposterior gradient that exists between the aqueous and vitreous humours. FGF-2 is the most important for lens epithelial cell proliferation and differentiation. It has been proposed that the presentation of FGF-2 to the lens epithelial cells involves the lens capsule as a source of matrix-bound FGF-2. Here we used immunogold labelling to measure the matrix-bound FGF-2 gradient on the inner surface of the lens capsule in flat-mounted preparations to visualize the FGF-2 available to lens epithelial cells. We also correlated FGF-2 levels with levels of its matrix-binding partner perlecan, a heparan sulphate proteoglycan (HSPG) and found the levels of both to be highest at the lens equator. These also coincided with increased levels of phosphorylated extracellular signal-regulated kinase 1 and 2 (pERK1/2) in lens epithelial cells that localised to condensed chromosomes of epithelial cells that were Ki-67 positive. The gradient of matrix-bound FGF-2 (anterior pole: 3.7 ± 1.3 particles/μm^2^; equator: 8.2 ± 1.9 particles/μm^2^; posterior pole: 4 ± 0.9 particles/μm^2^) and perlecan (anterior pole: 2.1 ± 0.4 particles/μm^2^; equator: 5 ± 2 particles/μm^2^; posterior pole: 1.9 ± 0.7 particles/μm^2^) available at the inner lens capsule surface was measured for the bovine lens. These data support the anteroposterior gradient hypothesis and provide the first measurement of the gradient for an important morphogen and its HSPG partner, perlecan, at the epithelial cell-lens capsule interface.

The eye lens comprises a lens capsule that encases a single layer of epithelial cells covering just the anterior hemisphere and the fibre cells that make the bulk of the lens. The lens grows throughout life through continuous epithelial cell proliferation and their differentiation into lens fibre cells at the lens equator. Fibroblast growth factors (FGFs) 1 and 2 regulate both lens cell proliferation and differentiation (McAvoy and Chamberlain, 1989), although of the two, FGF-2 is absolutely required (Garcia et al., 2005; Zhao et al., 2006). It, like FGF-1 is synthesized in the retina and ciliary body (Lovicu et al., 1997) and the FGFs are secreted into the aqueous and vitreous humours (Caruelle et al., 1989; Schulz et al., 1993), diffusing through the lens capsule where they can potentially bind to the heparan sulphate proteoglycan (HSPG) (De Iongh and McAvoy, 1992), perlecan (Iozzo, 1998; Tholozan et al., 2007). FGF-2 can be released from the capsule by matrix metalloproteinases (Tholozan et al., 2007) and bind to FGF receptors to activate the mitogen-activated protein kinase (MAPK) signalling cascade, resulting in the phosphorylation of MAPK1 (ERK1; Upadhya et al., 2013) that is localised in the nuclei of epithelial cells (Lovicu and McAvoy, 2001) effecting cell proliferation and differentiation (Golestaneh et al., 2004; Le and Musil, 2001; Lovicu and McAvoy, 2001) via specific transcription factors (Krishna and Narang, 2008).

Lens epithelial cell proliferation and differentiation are FGF-concentration dependent processes (McAvoy and Chamberlain, 1989). It is proposed that there is an anteroposterior gradient of FGF-1 and FGF-2 (Lovicu and McAvoy, 2005; Lovicu et al., 2011; McAvoy and Chamberlain, 1989), but so far this gradient at the cell-lens capsule interface has not been measured. Previous studies based on cross sections of rat lens capsules (Lovicu and McAvoy, 1993) have reported considerable variation in signal intensity with respect to anterior and posterior lens capsules, but the epithelial cell–capsule interface was not systematically investigated. Immunoblotting studies showed that the vitreous humour contains substantially more FGF-2 than aqueous humour (Schulz et al., 1993), but it must then traverse one of the thickest basement membranes in the body (Danysh and Duncan, 2009; Danysh et al., 2010) to reach the epithelial cell interface. Immunofluorescence microscopy reported the distribution of both FGF-2 and its ECM partner, perlecan in the lens capsule (Lovicu and McAvoy, 1993), but again the gradient was only qualitatively assessed. For mathematical modelling, the gradient of available morphogens such as FGFs (Bokel and Brand, 2013; Yu et al., 2009) and Hedgehog (Aguilar-Hidalgo et al., 2013; Nahmad and Stathopoulos, 2009; Su et al., 2007) in different aspects of eye development as well as Wnt in limb and wing development (Gao and Yang, 2013; Giraldez et al., 2002; Zecca et al., 1996) are all very important in their development and refinement.

In the present study, we have used immunogold labelling to measure FGF-2 and perlecan levels at the interface of the lens capsule with the epithelial cells. The whole lens capsule can be imaged once it is flat-mounted. This removes ambiguities about the relative position of the image and labelling inconsistencies within the sample. The immunogold signals for FGF-2 and perlecan on the lens capsule were then correlated with the location and degree of ERK1/2 phosphorylation in the central and peripheral lens epithelium.

At least three randomly selected regions from the equator, anterior and posterior poles of the inner surface of the bovine lens capsule were imaged (Fig. 1A). A secondary electron image (Fig. 1B) showing the capsule surface and corresponding backscatter electron image (Fig. 1C) showing gold particles were taken consecutively. A combined secondary/backscatter image was obtained at the end to show the location of gold particles on the lens capsule (Fig. 1D). The secondary electron images showed the compacted meshwork of the capsular surface free from cellular remnants, which is quite similar to a previous SEM study of lens capsular surface (Streeten, 1977). Collagen IV served as a positive control to confirm immunogold labelling efficiency because this has previously been shown to be an abundant protein in the bovine lens capsule by immunogold labelling (Cammarata et al., 1986). Many gold particles were present across the inner surface of lens capsule (Fig. 1E–G). The number at the anterior pole (287 ± 29/μm^2^) and lens equator (263 ± 52/μm^2^) was significantly higher than that measured for the posterior pole (140 ± 30/μm^2^; Fig. 1N) and comparable to previously reported labelling densities in the bovine lens capsule (Cammarata et al., 1986). The labelling density for FGF-2 (Fig. 1H–J) and perlecan (Fig. 1K–M) was much lower compared to collagen IV, but the labelling was specific compared to samples treated with secondary antibodies alone (data not shown). Labelling for both FGF-2 and perlecan were significantly higher at the lens equator (FGF-2: 8.2 ± 1.9 particles/μm^2^ [mean ± standard error of the mean]; perlecan: 5 ± 2 particles/μm^2^) than at either the anterior (FGF-2: 3.7 ± 1.3 particles/μm^2^; perlecan: 2.1 ± 0.4 particles/μm^2^) or posterior poles (FGF-2: 4 ± 0.9 particles/μm^2^; perlecan: 1.9 ± 0.7 particles/μm^2^) (*p* < 0.05) (Fig. 1O and P). This indicates for the first time that a gradient for matrix-bound FGF-2 and perlecan is present on the inner surface of the lens capsule.

Using freshly isolated lenses, the lens capsule and its associated lens epithelial cells were dissected into the central zone (CZ) and germinative zone (GZ) plus transitional zone (TZ) (Fig. 2A). This dissection was based on our previous Ki-67 staining data that revealed the GZ location. Whole cell protein extracts were prepared from the lens epithelial cells at these capsule locations. Immunoblotting results showed that strong signals for the total and phosphorylated ERK1/2 (pERK1/2) were present in the cells from the CZ and GZ + TZ (Fig. 2B) again in agreement with previously published data for human (Maidment et al., 2004) and animal lenses (Golestaneh et al., 2004; Iyengar et al., 2006; Le and Musil, 2001; Lovicu and McAvoy, 2001). The pERK1/2 was 30% higher in the GZ + TZ compared to the CZ (Fig. 2C), indicative of increased flux through this signalling pathway and as evidence that the higher labelling of FGF-2 at the lens capsule interface could be correlated with this increased activity. This regional difference of pERK1/2 levels in bovine lens epithelium complements previous studies in human (Maidment et al., 2004) and rat lenses (Iyengar et al., 2006). Phosphorylated ERK1/2 levels were reported to be higher at the equator than the CZ of the human lens (Maidment et al., 2004), but in the rat, both regions have equivalent levels (Iyengar et al., 2006). If it assumed that pERK1/2 levels correlate with the proliferation index in the lens epithelium, then the latter does decrease with age (Wiley et al., 2010) and the ages of the rats (10 days), cows (<6months) and humans (adults, age unspecified) were not comparable. Others have shown that MAPK signalling is not uniform across the lens epithelium (Dawes et al., 2013; Le and Musil, 2001; Lovicu and McAvoy, 2001, 2005; Maidment et al., 2004) with ERK1 (MAPK1) being the dominant activity within the GZ (Upadhya et al., 2013). In the present study, only a few cells in the GZ had a distinctive nuclear pERK1/2 signal by immunofluorescence microscopy (Fig. 2D–G). This pattern differed to that previously reported for neonatal rats (Lovicu and McAvoy, 2001) where all cells in the epithelium showed punctate nuclear pERK1/2 labelling. Our studies show that the nuclear staining in the selected bovine lens epithelial cells was punctate and it localised to the surfaces of condensed chromosomes. This represents a novel finding for lens epithelial cells. Co-staining with Ki-67 and DAPI staining confirmed that it was only the epithelial cells at the G2/M or M phase of the cell cycle that were stained with pERK1/2. Importantly these were localised in the GZ region as further evidence of the functional link between nuclear-located pERK1/2 and lens epithelial cell proliferation. These observations also resonate with the reported kinetochore location of pERK1/2 in other proliferating cell systems (Shapiro et al., 1998; Willard and Crouch, 2001; Zecevic et al., 1998). This suggests that pERK1/2 may play a role in microtubule capture and chromosome condensation in early and mid-mitotic stages also in lens epithelial cells.

Our quantitative immune-electron microscopy data confirm that there is indeed a gradient of available FGF-2, with at least 2–2.5 times more FGF-2 available at the lens equator than at either the anterior or posterior poles on the inner surface of the lens capsule (Fig. 1). These data advance the earlier qualitative immunofluorescence-based study of FGF-2 staining in the capsule lamina (Lovicu and McAvoy, 1993) by quantifying the FGF-2 gradient across the inner surface of the bovine lens capsule.

Such information can be used to develop predictive models of epithelial tissues, because the morphogen gradient is an important parameter (Smith et al., 2012; Swat et al., 2012). We have measured the FGF-2 gradient available to the lens epithelium at the capsule interface. This FGF-2 will likely be bound to perlecan in the lens capsule, which not only protects FGF-2 from degradation, but also regulates its availability to the lens epithelial cells (Evans et al., 2002; Tumova and Bame, 1997). HSPGs are also established regulators of morphogen gradients (Giraldez et al., 2002), but FGF-2 can also influence HSPG processing (Sasisekharan et al., 1997; Tumova and Bame, 1997; Tumova et al., 1999) and can also increase HSPG synthesis (Evans et al., 2002; Mashayekhi et al., 2011) as a feedback loop that could further regulate the levels of FGF-2. Such issues have yet to be investigated for the lens capsule.

Central to the FGF gradient hypothesis is the anteroposterior distribution of FGF-1 and 2 (Lovicu and McAvoy, 2005; Lovicu et al., 2011; McAvoy and Chamberlain, 1989). Levels of FGF-2 are higher in the vitreous humour compared to the aqueous humour (Schulz et al., 1993), but the levels present on the posterior lens capsule are significantly lower than the lens equator and are most similar to the lens anterior surface. Flow models for the aqueous (Fitt and Gonzalez, 2006) and the vitreous (Bonfiglio et al., 2013) humours can potentially explain why in the anterior segment, the concentration gradient of FGF-2 declines towards the anterior pole and why in the posterior segment it also declines toward the posterior pole, remembering that the two chambers are separated by the hyaloid membrane (Snead et al., 2002). The coincidental distribution in available perlecan epitopes and therefore potential HSPG docking sites for sequestering FGFs we suggest is an equally important factor and is reported for the first time here.

## Figures and Tables

**Fig. 1:**
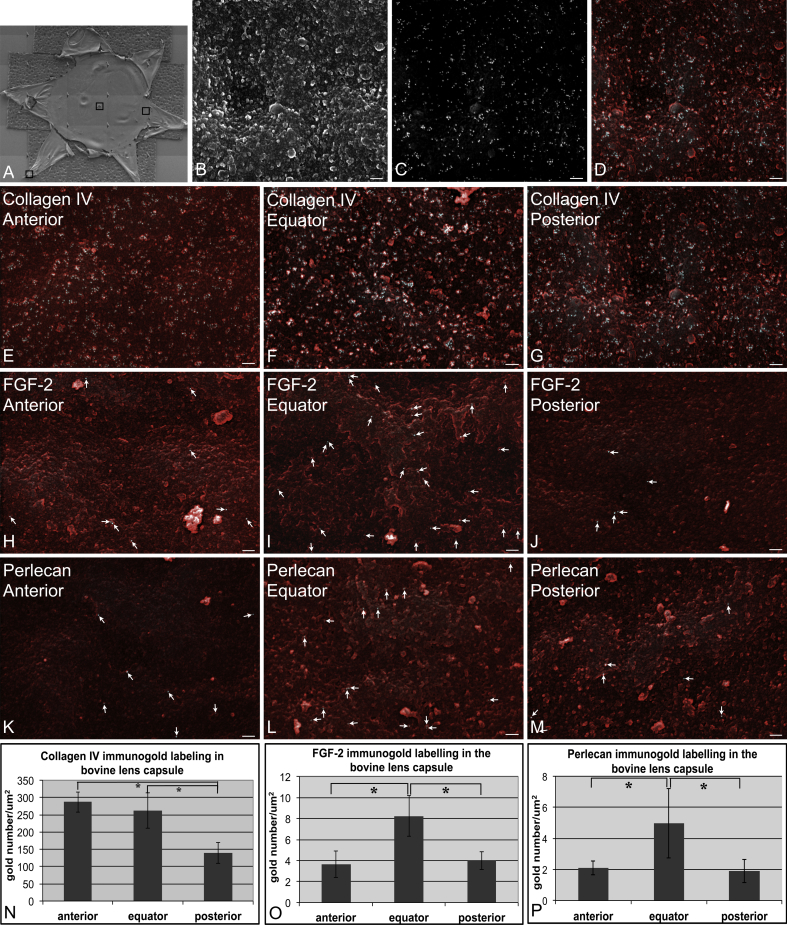
Measurement of FGF-2, collagen IV and perlecan at the epithelial cell – lens capsule interface by immunoelectron microscopy. Bovine lens capsules were flat-mounted and washed in deionised water for one hour to remove epithelial cells. They were then incubated with either rabbit anti-collagen IV polyclonal antibody (Abcam, UK), or rabbit anti-FGF-2 polyclonal antibody (Calbiochem, USA) or mouse anti-perlecan monoclonal antibody (Chemicon International, USA) for 2 h at room temperature, followed by an hour of incubation with the appropriate secondary antibodies (either goat anti-rabbit or goat anti-mouse IgG both conjugated with 10 nm gold; BBInternational, Cardiff, UK). Negative controls were incubated with PBS instead of primary antibodies. Immunogold labelled capsules were viewed in a Hitachi SU-70 FEG scanning electron microscope (Hitachi Hight-Technologies Europe GmbH, Germany). Each labelling was independently repeated three times. A. An example of a lens capsule prepared for scanning electron microscopy. Three images were then randomly taken at the equator and the anterior and posterior poles (black squares). B. The secondary electron image shows the capsular surface and its compacted meshwork. C. A backscatter electron image of the same area as in panel B shows a large number of white gold particles that indicate collagen IV labelling. D is the combined secondary/backscatter image of panels B and C to show the location of gold particles on the lens capsule. E–M: Representative images show the immunogold labelling of collagen IV (E–G), FGF-2 (H–J) and perlecan (K–M) in different regions of the inner surface of the bovine lens capsule. More gold particles were present after collagen IV labelling than after FGF-2 and perlecan labelling (white arrows). N–P: The quantification of gold particles detected on lens capsules after Collagen IV, FGF-2 and perlecan labelling. Significant differences tested by Student's *t*-Test are shown (*). B–M. Scale bars = 100 nm.

**Fig. 2:**
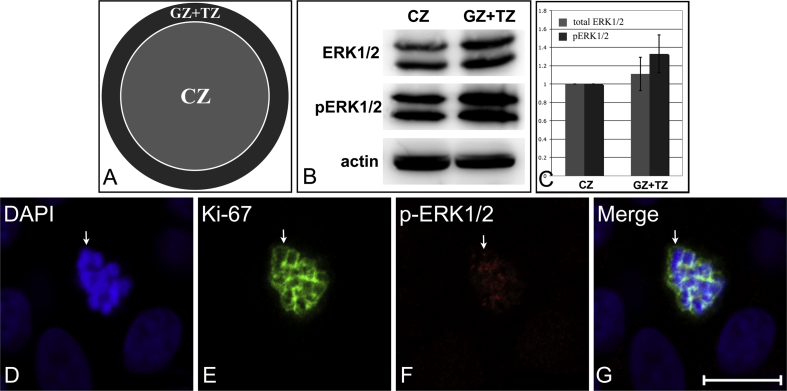
Regional and subcellular distribution of pERK1/2 in the bovine lens epithelium. A. A diagram to show the dissection of a lens epithelium for immunoblotting. B. Representative immunoblotting bands of lens epithelial cells in the CZ and GZ + TZ assayed for total ERK1/2, phosphorylated ERK1/2 and actin. Total cell proteins in the CZ and GZ + TZ were extracted and 10 μg was analysed by immunoblotting with mouse anti-ERK1/2 monoclonal antibody, rabbit anti-phospho-ERK1/2 monoclonal antibody (Thr202/Tyr204) (both from cell signalling, USA), and mouse anti-β-actin antibody (MP Biolmedicals, LLC., USA). C. Quantification results of the immunoblotting signals. The ERK1/2 and pERK1/2 signals were quantified and the relative band density was obtained using the formula based on previous reports (Wang et al., 2010; Wang et al., 2009): (*D*_ERK1/2_/*D*_β-actin_)_GZ+TZ_/(*D*_ERK1/2_/*D*_β-actin_)_CZ_ and (*D*_pERK1/2_/*D*_β-actin_)_GZ+TZ_/(*D*_pERK1/2_/*D*_β-actin_)_CZ_, where *D* represents the band density. *D*_ERK1/2_/*D*_β-actin_ adjusts the density of each band against standard band β-actin. This experiment was independently repeated three times. By the two tailed Student's t-test, the difference between pERK1/2 levels in the CZ and GZ+TZ was 0.051. D–G. A representative mitotic cell labelled with pERK1/2. The flat-mounted bovine lens epithelium was stained with pERK1/2, Ki-67 (Dako, Denmark) and DAPI. Images with pERK1/2-positive cells were taken using a Zeiss LSM 510 Meta scanning confocal microscope (Carl Zeiss Inc., Jena, Germany). The DAPI (D) and Ki-67 (E) labelled condensed nuclear chromosomes suggest that this cell is in M phase. Punctate phosphorylated ERK1/2 staining is distributed along the chromosomes (F). Scale bar = 100 μm.
